# A Pill for the Ill? Patients’ Reports of Their Experience of the Medical Encounter in the Treatment of Depression

**DOI:** 10.1371/journal.pone.0066338

**Published:** 2013-06-18

**Authors:** Andreas Vilhelmsson, Tommy Svensson, Anna Meeuwisse

**Affiliations:** 1 Nordic School of Public Health, Gothenburg, Sweden; 2 Department of Behavioural Sciences and Learning, Linkoping University, Linkoping, Sweden; 3 School of Social Work, Lund University, Lund, Sweden; Charité University Medicine Berlin, Germany

## Abstract

**Background:**

Starting in the 1960s, a broad-based patients’ rights movement began to question doctors’ paternalism and to demand disclosure of medical information, informed consent, and active participation by the individual in personal health care. According to scholars, these changes contributed to downplay the biomedical approach in favor of a more patient-oriented perspective. The Swedish non-profit organization Consumer Association for Medicines and Health (KILEN) has offered the possibility for consumers to report their perceptions and experiences from their use of medicines in order to strengthen consumer rights within the health care sector.

**Methodology:**

In this paper, qualitative content analysis was used to analyze 181 KILEN consumer reports of adverse events from antidepressant medications in order to explore patients’ views of mental ill health symptoms and the doctor-patient interaction.

**Principal Findings:**

Overall, the KILEN stories contained negative experiences of the patients’ medical encounters. Some reports indicated intense emotional outrage and strong feelings of abuse by the health care system. Many reports suggested that doctors and patients had very different accounts of the nature of the problems for which the patient was seeking help. Although patients sought help for problems like tiredness and sleeplessness (often with a personal crisis of some sort as a described cause), the treating doctor in most cases was exceptionally quick in both diagnosing depression and prescribing antidepressant treatment. When patients felt they were not being listened to, trust in the doctor was compromised. This was evident in the cases when the doctor tried to convince them to take part in medical treatment, sometimes by threatening to withdraw their sick-listing.

**Conclusions:**

Overall, this study suggests that the dynamics happening in the medical encounter may still be highly affected by a medical dominance, instead of a patient-oriented perspective. This may contribute to a questionable medicalization and/or pharmaceuticalization of depression.

## Introduction

Ever since the 1970s the medical encounter has been under sociological investigation, which has revealed conflicts and tensions that arise as patients and their doctors negotiate and bargain over aspects of care [1]. Starting in the 1960s, patients’ rights movements began to question the authority of doctors and demand informed consent and disclosure of medical information [2]. They criticized traditional doctor-patient communication for not including a role for patient health beliefs [3]–[4] and for neglecting patients’ priorities and concerns [5]. According to scholars, this development contributed to downplaying the biomedical approach of modern health care in favor of a more patient-oriented perspective [1].

Parallel to this development, psychiatry in the 1970s imported the diagnostic model from medicine to replace a more dynamic model [6] and since the 1980s (and the release of the *Diagnostic and Statistical Manual of Mental Disorders*, 3^rd^ edition [DSM III]) increasingly has started to embrace a biological orientation [7]. The advent of psychotropic drugs is believed to have given rise to a new biological language in psychiatry [7]. The biomedical approach is often referred to as the model of modern medicine, or the ‘medical model’. Proponents of this model view disorders as having physiological/anatomical foundations and prescribe physiological/anatomical treatment [8]. The medical model was highly contested in the 1970s, when critics like Ivan Illich [9] and Irving Zola [10] (among others) highlighted medicalization as an increasing problem for the society. As Peter Conrad, for example, argued, medicalization occurs when a medical frame or definition is applied to understand or manage a problem previously not considered a medical problem [11]–[12]. Medicalization represented a fundamental shift in thinking among medical sociologists by highlighting the potential inequity taking place in medical encounters [13]; it was an alternative way to understand the dynamics between doctor and patient [14].

Depression is now considered a growing burden for society and a significant public health concern across all regions of the world. The World Health Organization (WHO) even predicts depression to be a main contributor to the global disease burden by 2020 [15] and 2030 [16]. According to some scholars, the increasing numbers of diagnoses of depression, and the ensuing prescriptions of antidepressants to treat it, reflect two concurrent phenomena: the ‘medicalization of distress’ and a growing view that depression is primarily a ‘neurochemical disorder’ that can be corrected with a drug [17]. It has also been claimed that antidepressants reflect one of the major manifestations of the medicalization of modern society [18].

Drug dependency and concern about potential overdosing (mostly barbiturates and benzodiazepines) started to be acknowledged and taken seriously in the 1960s and 1970s and have continued to be seen as important [19]–[20]. In Sweden, this development resulted in the creation of non-profit organizations like the National Association for Aid to Drug Abusers (RFHL) in 1965 and the Consumer Association for Medicines and Health (KILEN) in 1992. In 1997, KILEN established a consumer database in order to collect consumer reports that focused mainly on adverse events and adverse drug reactions (ADRs) from benzodiazepines and antidepressants. These reports constitute unique consumer reporting material in Sweden. Since 2002, it has also been possible to report experiences with medicines to KILEN through a web-based report form (www.kilen.org). It has been argued that many patient reporting systems focus only on adverse events and risk missing other aspects of medicine use like experiences of ineffectiveness [21]. With the web-based report form provided by KILEN, however, it is also possible to add free text comments of the experience(s). KILEN as a consumer institute was unexpectedly forced to cease operations in March 2007, when the Swedish Parliament (Riksdag) decided not to allow further government grants [22]–[23]. Despite these changes, it is still possible to report adverse events and ADRs through the web-based report form.

Earlier studies of the KILEN material have indicated that consumer reports might contribute valuable information regarding more serious psychiatric ADRs following antidepressant treatment [24] and that free-text comments can provide important information on how a drug may affect the person using it and influence his or her personal life [25]. The aim of this study was to explore patients’ views of mental ill health symptoms and their experiences of the doctor-patient interaction as they expressed them in the KILEN reports.

## Methods

All reports of suspected adverse reactions regarding antidepressant medications submitted from January 2002 to April 2009 to KILEN’s Internet-based reporting system in Sweden were analyzed according to reported narrative experience(s). An ADR is defined as a response to a medicine that is noxious and unintended and that occurs at doses normally used in humans, whereas an adverse event or experience is defined as any untoward medical occurrence that may present itself during treatment with a medicine but that does not necessarily have a causal relationship with this treatment [26]. A report in the KILEN material was equal to one individual’s reported experience with a drug and an ADR was equal to one single reported effect connected to a specific drug. More than one ADR related to the same drug could be submitted. The reported ADRs to KILEN were compiled and coded in a similar way to those listed in the Swedish Physicians’ Desk Reference. KILEN personnel accomplished this by using the database software FileMaker. The regulatory authorities like the Medical Products Agency do not handle data submitted to KILEN. Of 442 individual antidepressant reports, 393 individuals also provided a longer description of their adverse experiences as free text (89%). A total of 202 antidepressant reports concerned depression as a diagnosis (the most reported cause for prescription) and included a narrative of the experience(s) (46%). A total of 21 reports were excluded because they were reported by someone other than the patient (5) or contained too little information (16). Included in the study, therefore, were 181 reports (41%) with narrative.

### Data Analysis

Patients’ accounts were interpreted using qualitative content analysis. Content analysis here refers to a qualitative data reduction and sense-making effort that takes a volume of qualitative material and attempts to identify core consistencies and meanings [27]. The procedure is as follows: data are collected and coded by theme or category; the coded data are then analyzed and presented [28]. Creating categories is the core feature of qualitative content analysis and refers to a descriptive level of content; a category often includes a number of sub-categories [29]. All 181 included consumer narratives on depression and antidepressant treatment were read thoroughly several times in order to get an understanding of their content. The content of these narratives was then sorted into different main categories and read again, which resulted in subcategories and sometimes new main categories. Content analysis involves a balancing act, where on one hand it is impossible and undesirable for the researcher not to add a particular perspective to the phenomena under study, but on the other hand the researcher must ‘let the text talk’ and not impute meaning that is not there [29]. Therefore, all authors were involved in analyzing the themes that emerged from the data and were responsible for reading and confirming the analysis. The authors discussed the analyses – the coding, categorization, and interpretation of the results – throughout the work process to gain a mutual understanding. This process was valid also for the selection of quotations describing common experiences found within certain categories. This selection also was made in order to problematize the role of the researcher and to help the researcher avoid missing vital information or exaggerating specific content.

### Methodological and Ethical Considerations

The KILEN data material was based on spontaneous consumer reports and thereby was selected material, which might have exaggerated a negative view and experience of the medical encounter. It is therefore unlikely that all views and experiences of the doctor-patient interaction have been captured. Because it is an Internet-based reporting system, it most likely will benefit younger individuals who are used to handling a computer, but by missing the older age groups’ experiences, one risk getting a biased view of patients’ experiences of treatment. A Danish study showed, for instance, that older female patients with depressive disorder had more negative views of the doctor–patient interaction and of antidepressants [30]. We must also acknowledge that data were recorded between 2002 and 2009, so some patients’ experiences of the medical encounter may be older than 2002 and some reports refer to older guidelines in health care. There is also the issue of gender. Previous studies have indicated that women reported adverse events to KILEN in a much higher proportion: between three and four times more often than men, and sometimes more within certain age groups [24]–[25]. This may be an effect of women turning to non-profit organizations for help possibly to higher degree. It may also be an effect of women tending to have a higher risk of adverse events than men; effects that increase with age and number of drugs prescribed [31], and also could explain women’s over-representation in reporting to KILEN. Furthermore, we do not know how consumers/patients were ‘officially’ diagnosed with depression (ICD-10, DSM-IV or other), and we do not know if the reported diagnosis was a ‘valid’ one, because we have only the patients’ own reported experiences to the KILEN website. It is also important to acknowledge that this was only the patients’ perception of the medical encounters, so we cannot compare doctors’ perceptions. Although the important information from the narrative reports stands as valid for those who reported, there is not a denominator to provide perspective about the frequency of such experience. Trustworthiness is crucial when performing qualitative research [32]. The number of patient narratives and all researchers cross-checking the data material should strengthen trustworthiness. Despite the limitations of this study, the data are of value because the material provides unique information about consumer reporting (in Sweden) and patients’ qualitative experiences of the doctor-patient relationship in the treatment of depression.

The Declaration of Helsinki aims to ensure that research is carried out in an ethical way and follows accepted scientific principles [33]. According to the Council for International Organizations of Medical Sciences (CIOMS), all research involving human subjects should be conducted in accordance with three basic ethical principles: respect for persons, beneficence, and justice [34]. These ethical guidelines were followed throughout the study. Reporters were informed that their voluntary submission of adverse event reports through the KILEN website the material could be compiled and used for research but that no personal information would be identified. Reporters were also given the chance to provide information anonymously. Written consent was for practical purposes not collected, but informants were informed that they could withdraw their report or withhold their consent for scientific publication by contacting the organization. Furthermore, the database manager at KILEN coded the material and made it anonymous by removing the reporters’ names and residences and replacing them with a number.

The Regional Ethics Review Board in Gothenburg, Sweden, approved the project (No. 319-10). The ethics committee approved the consent procedure.

## Results and Discussion

Of the 181 consumer reports included and analyzed, 81 contained a qualitative description of the medical encounter (women 81% and men 19%). As described in Table 1, three main categories emerged from the analysis of the KILEN data: (1) *different interpretation and understanding of the problem*, (2) *choice of treatment strategy,* with subcategories (a) *antidepressants as the obvious choice* and (b) *psychotherapy seldom an alternative,* and (3) *trust and distrust* with subcategories (a) *experiencing indifference and nonchalance* and (b) *feeling forced to accept diagnosis and treatment,* and (c) *feeling abandoned by the doctor.*

**Table 1:** Categorization of the analyzed components – examples of patients’ statements in the KILEN consumer reports.

Meaning unit	Condensed meaning unit	Main-category	Sub-category
*In fact, my so-called ‘depression’ was a* *normal reaction to crisis following* *separation, homelessness, loss of two* *jobs within three years, and death in the family.*	The physician diagnoses depressionwhile the patient thinks it is a normalreaction to life events.	Different interpretations and understandings of the problem	
*The doctor has told me to continue in order* *to feel better and that I shall* *understand it as a ‘vitamin boost’*	The patient experience that the doctorcompares antidepressants to vitaminsso that she will stay on them	Choice of treatment strategy	Antidepressants as the obvious choice
*All I wanted was someone to talk to,* *some sort of therapy.*	The patient wants therapy.		Psychotherapy seldom an alternative
*The first doctor I visited barely looked at* *me when I told her about my symptoms*	The patient feels that the doctoravoids eye contact when she is tryingto describe her symptoms.	Trust and distrust	Experiencing indifference and nonchalance
*…I refused despite threats of ending my* *sick-listing, since I ‘apparently did not* *want to get better as I was avoiding* *work’, as he [the doctor] concluded.*	The patient is feeling threatened bythe doctor to accept diagnosis.		Feeling forced to accept diagnosis and treatment
*While I have been medicating my* *doctor and I have not spoken*.	The patient feels being left adrift bythe doctor		Feeling abandoned by the doctor

### Different Interpretation and Understanding of the Problem

A central theme concerned patients’ and doctors’ different interpretations and understandings of the presented problem in the medical consultation. Many patients did not explicitly mention their experience and understanding of the issue for which they sought medical attention, but approximately 20% of the patients reported going to a doctor with a non-specific understanding. However, some patients were hesitant to accept an immediate medical understanding of the problem. These patients often had their own notions as to what had caused their problems, usually referring to stress or traumatic changes in their personal life situations. This could include previous medical problems (for instance, cancer treatment), problems at work or losing a job, but also the loss of a loved one. One woman described her depression as a normal reaction to a problematic life situation.

*In fact, my so-called ‘depression’ was a normal reaction to crisis following separation, homelessness, loss of two jobs within three years, and death in the family.* (Woman, 63 years old).

Some patients reported not having the strength to argue with their doctor’s decisions and instead agreed on the diagnosis presented to them (in this case depression). A few patients reported that they protested against a medical understanding of their problem but that the doctor then further stressed it as a medical one, for instance, by equating all fatigue-like states with depression.

*Went to see a doctor because I was exhausted. Could not sleep, could not think, had stopped working. The doctor said it was depression, but I was hesitant. I did not feel depressed, just tired and sad about the terrible situation I was in…He stated all symptoms of fatigue to be the same as depression.* (Woman, 41 years old).

Previous qualitative studies have shown that doctors interpret depression differently than patients do [35]–[36] and that doctors often fail to recognize the social context of depression [37]. Some scholars argue that a problem with the biomedical model is that it makes patients’ stories increasingly irrelevant to treatment [38], reducing the experience of depression to a clinical target [39]. In the early 1990s psychiatrist Peter Kramer acknowledged in his landmark book *Listening to Prozac* how the (at the time) new antidepressant SSRI (selective serotonin reuptake inhibitor) drug changed his way of thinking about the inner human mind, away from a psychological process of thinking towards a more biological model, where symptoms lack social meaning [40]. It is important to recognize, however, that doctors alone are not to be held responsible. They use their medical knowledge and language (as they are trained to do), but all too often they lack the time needed for a more thorough examination of the patient. The adopted strategies also may be related to savings and cutbacks or changed guidelines within the health care system affecting both doctor and patient. Medical encounters take place within a system where diagnostic handbooks and short-form tests are used as a fast way of judging a person’s health status, a system that allows and encourages doctors to swiftly choose a diagnosis without a comprehensive investigation of the whole situation surrounding the patient. The Swedish National Board of Health and Welfare has indicated, for instance, that there are deficiencies regarding how psychiatric conditions are diagnosed and documented, which can contribute to both overtreatment of some patients and undertreatment of others [41]. However, it was not only doctors who interpreted patients’ symptoms in biomedical or psychiatric terms in the KILEN reports. A few patients seemed quite familiar with medical language.

*I have had a very severe, lonely, and anxious childhood (not because of incest or physical violence) and as an adult have had more and more frequent and deeper periods of apathy and depression. My memory works poorly, and I have had big blackouts in the past and have needed therapy to make out what is missing.* (Woman, 34 years old).

According to scholars like Nikolas Rose, people increasingly have come to understand themselves as shaped by their biology [42]; medicalization has made medicine inextricably intertwined with the ways in which individuals experience and give meaning to the world [43]. One risk with this development is that patients who feel well may become symptomatic because they are told there is something wrong with them [14]. It is important to acknowledge, however, that medicalization can benefit patients by giving their condition attention and treatment. The KILEN narratives imply that most of the individuals who sought help within the health care system indeed shared the notion that they had some sort of mental ill health problem requiring professional help. Hence, one could argue that these individuals in a way medicalized themselves.

### Choice of Treatment Strategy

A second main category that emerged from the analysis concerned the treatment offered to deal with patients’ issues. According to several consumer reports a medical diagnosis was rapidly formalized with a subsequent decision about medical treatment.

#### Antidepressants as the obvious choice

Some of the patients perceived that a prescription of antidepressants was issued without the doctor asking for or listening to their story. Some patients had experienced this with more than one doctor. Antidepressant drugs were occasionally offered during the first consultation, and sometimes even in the beginning of this meeting.

*I was not feeling well after my second breast cancer and was offered psychiatric help and thought that it would be useful to talk to someone, but after twenty minutes, first consultation, I was offered ‘happy pills’.* (Woman, 50 years old).

This has been recognized in earlier qualitative research as well, where patients were prescribed antidepressants during their first visit to their doctor for depression [44]. Even though several patients reported expressing their concern about taking an antidepressant drug, some of them perceived that their views were not taken into account when the doctor was deciding on treatment options. This was particularly evident amongst those patients who reported being afraid of taking drugs in general and antidepressants in particular. A few patients reported having negative experiences of this kind of treatment in the past.

*I do not like taking pills and told this to the doctor. Then she proposed Valium [Swedish benzodiazepine brand name (substance: Diazepam) – author’s not] so I would feel more relaxed in taking Seroxat [Swedish antidepressant brand name, substance: Paroxetine – author’s note].* (Woman, 50 years old).

The antidepressant treatment strategy, according to some patients, was often or nearly always issued within a medical understanding of what depression is and how antidepressant treatment works. According to patients’ statements this sometimes meant that doctors used familiar metaphors to which patients were supposed to be able to relate. Antidepressant drugs were compared with vitamin pills in one case, as something providing energy

*The doctor has told me to continue in order to feel better and that I shall understand it as a ‘vitamin boost’.* (Woman, 36 years old).

Previous qualitative studies have shown that doctors often made comparisons with diabetes in order to simplify the role of antidepressants in depression [37] and that patients themselves even compared antidepressants to vitamins [45]. The analogy of depression was presented in some cases as a chemical imbalance that the antidepressant would correct. One patient expressed doubt about this analogy.

*Maybe the root cause is not a chemical imbalance in the brain!* (Woman, 38 years old).

This problem also has been suggested in previous research, where doctors told their patients that antidepressants would correct a ‘chemical problem in their nervous systems’ [37] or that SSRIs would address ‘an imbalance in the brain’ [46]. Several patients in the KILEN material reported being on antidepressants for many years, and a few patients had been informed that the treatment was not something temporary, but instead could be life-long treatment. For some of them the antidepressant drug therapy was presented as a solution that would compensate for a shortage of something lacking in the patient’s body, in this case serotonin in the brain.

*I along with my doctors know that I have low levels of serotonin and one doctor told me that I probably will have to take Cipramil [Swedish antidepressant brand name (substance: Citalopram – author’s note] for the rest of my life* (Woman, 38 years old).

The understanding of depression as a biochemical disturbance in the brain has progressed from theories introduced in the mid-1960s by Joseph Schildkraut in 1965 [47] and Alec Coppen in 1967 [48] to become particularly influential. These theories had a considerable impact on the course taken by research in psychiatry, neuropharmacology, psychopharmacology, and neurochemistry [49]. Depression was no longer seen as just a natural response to stress; there was now an underlying biological factor, which was the ‘cause’ of depression [50]. Nevertheless, the understanding of a chemical imbalance is disputed [7], [40], [51]–[56], and it is argued that there is no scientifically established ideal of a ‘chemical balance’ of serotonin, let alone an identifiable pathological imbalance [18].

Some patients in the KILEN material reported that it felt like antidepressant medication was all the doctors had to offer, that there were no alternatives presented to them. Either they took the pills offered or there was nothing the doctor could do for them.

*I have felt that the reason for doctors to prescribe antidepressant medication is that it is the only help they can offer, and that this is why the doctor can be frustrated if you reject this help.* (Woman, 38 years old).

This has been recognized during earlier qualitative research, where patients perceived that professionals had nothing more than pills to provide for depressive symptoms [36]. Some scholars even argue that we now can speak of a ‘pharmaceuticalization’ of everyday life as the pharmaceutical industry introduces profitable medicines for a range of daily activities and where pharmaceuticals are understood by consumers as ‘magic bullets’ to solve problems of everyday life [57]. Pharmaceuticalization is by one writer defined as *“the process by which social, behavioral or bodily conditions are treated, or deemed to be in need of treatment, with medical drugs by doctors or patients”* [58]. This would imply that because depression already is a medical condition (medicalized), the increasing consumption of antidepressants would suggest a pharmaceuticalization of the condition. The expansion of the pharmaceutical market is suggested therefore to be a vital aspect of pharmaceuticalization [58]. Global pharmaceutical sales have increased from $500 billion in 2003 to $856 billion in 2010 [59], with global sales of antidepressants for more than $20 billion (ranked ninth among prescription drugs) [60]. In the absence of therapeutic alternatives, the SSRIs are projected to continue to dominate the antidepressant market to 2018 and to increase its sales from $11.9 billion in 2011 to $13.4 billion [61].

It is sometimes argued that the widespread use of antidepressants has helped to reinforce the idea that personal problems could be attributed to a chemical imbalance [62]–[63], leading to potentially unjustified pharmaceuticalization. For instance, pharmaceutical advertising, especially direct-to-consumer (DTC), may encourage healthy people to think they need medical attention [64]. In the United States, DTC advertising campaigns of SSRIs have largely revolved around the claim that the drug corrects a chemical imbalance caused by a lack of serotonin [18]. Direct advertising to consumers is not allowed in Sweden but is done indirectly through doctors. DTC advertising has been accused of medicalizing the human experience [65] and tends to drown out public health messages about individual factors like diet and exercise, for example, and to ignore bigger societal issues like social involvement and equity [66].

#### Psychotherapy seldom an alternative

According to some KILEN narratives, psychotherapy was seldom presented to them as a valid treatment option, despite patients sometimes requesting it, usually with a belief that they needed someone to talk to about their issues.

*All I wanted was someone to talk to, some sort of therapy.* (Woman, 22 years old).

These patients were often convinced of the value of psychotherapy (often cognitive behavioral therapy, CBT) and appeared to be quite familiar with the treatment. A few patients turned to private caregivers just to be certain they would get the treatment they wanted. Patients who were offered psychotherapy (psychotherapy alone or in combination with antidepressants) reported being more satisfied. It is important to acknowledge, however, that patients’ desires for wanting ‘someone to talk to’ in terms of psychotherapy can also mean that social problems are being medicalized.

Previous research has shown that it appears that doctors are less willing to consider nondrug treatments if drug therapy is an available option [67]. Furthermore, if drugs are the only form of therapy being publicized through ads, seminars, and other publicity, the chances are slim that alternative modalities, such as psychotherapy, will be used [68]. The act of prescribing in itself might also suggest a biological basis for a problem [69]. Some patients’ stories to KILEN indicated that a prescription was certainly not what the patient had in mind. Doctors using a prescription as a substitute for time or as a coping strategy have also been described in previous research [70]–[71]; patients were neither satisfied nor enabled when handed nothing but a prescription [72]. According to the WHO, health care providers should not passively consider medications as their only therapeutic strategy, and patients should not be given a message suggesting that modifications of thought, mood, and conduct can be achieved by pharmacological means only [73].

Ghostwriting is another problematic issue in drug treatment [55]–[56], [58]. This refers to academic articles that are written covertly by a commercial writer employed by a pharmaceutical company; the articles carry an academic’s name on it to give it the impression of independence and scientific rigor [74]. A study from 2011 showed, for instance, that 7.9% of the papers in six leading medical journals were ghostwritten [75]. This practice may foster an agenda where pharmaceutical companies write scientific articles in order to promote a certain drug treatment for a medical condition. In recent years it has also been revealed that members of the panels for DSM-IV and DSM-5 have financial ties to the pharmaceutical industry [76]–[77]. There is also the issue of non-publication of trials or exclusion of relevant data from published trials, risks leading to inaccurate recommendations for treatment [78]. Selective reporting (for example, publishing more favorable results for per protocol population when the prespecified population for analysis had been the intent to treat population, or vice versa) has been shown to be a major cause for bias, implying that any attempt to recommend a specific SSRI from the publicly available data is likely to be based on biased evidence [79]. Thus, we must be aware of selective publication that can lead doctors to make inappropriate prescribing decisions that may not be in the best interest for either patients or public health [80].

### Trust and Distrust

In a third main category, some patients referred to losing trust in their doctor when they perceived that he or she did not care about them as patients and/or did not acknowledge their reasons for seeking help in the first place. Trust was sometimes compromised as early as in the first consultation.

#### Experiencing indifference and nonchalance

Some of the narratives contained experiences of arrogance and an unsympathetic attitude from the doctor. This could mean that a patient felt the doctor misunderstood or did not take him or her seriously during the communication. Several patient narratives included experiences of the doctor’s indifference and neglect when the patients were describing their symptoms.

*The first doctor I visited barely looked at me when I told her about my symptoms.* (Woman, 42 years old).

Patients’ expressions of indifference in the KILEN material included not only doctors but sometimes the entire health center, where some patients expressed that they were not offered any help at all. In some cases, however, patients reported trusting their doctor if he or she was a specialist, usually a psychiatrist. A few patients even argued that the general practitioners did not have the knowledge required to prescribe antidepressant medication. Those who reported being offered a specialist argued that they as patients had something to say regarding diagnosis, content, and treatment and, above all, a right to be listened to.

*I have the ‘luck’ nowadays of having ongoing contact with psychiatrists with solid knowledge of the field and who also order laboratory tests to ensure that the right medicine is prescribed.* (Woman, 49 years old).

Patients’ trust in doctors has been emphasized in earlier research as extremely important [3], [81] and is usually associated with patients’ perceptions of doctors’ medical expertise and capability [62]. It has also been suggested that doctors can contribute to a more beneficial consultation by showing responsiveness towards and respect for the patient [14] and by listening to and acknowledging the patient’s own understanding of ill health [36].

#### Feeling forced to accept diagnosis and treatment

Patients’ trust in their doctors was further diminished when they perceived that the doctor tried to force them to accept a diagnosis and antidepressant medication as a condition of receiving any treatment at all or as a prerequisite for sick-listing.

*After a couple of months of being sick-listed because of severe burnout, the doctor decided to issue an ultimatum: either I started with Fluoxetine [Generic antidepressant, substance: Fluoxetine – author’s note], or he would not continue my sick-listing.* (Woman, 26 years old).

Eliot Freidson described early professional dominance as the phenomenon of subordination of the layperson’s perspective to the professional perspective [3], [82]. In essence, the process of treatment and care may be seen as a process that attempts to influence the patient to behave in the ways considered appropriate to the illness that has been diagnosed, a process often called ‘management by professionals’ [3]. Some patients reported giving in to antidepressant medication as a way of securing their rights to sick-listing. A few patients reported not being able to discontinue antidepressants medication because this would terminate their sick-listing. One patient even described being accused of not wanting to improve.

*…I refused despite threats of ending my sick-listing, since I ‘apparently did not want to get better as I was avoiding work’, as he [the doctor] concluded.* (Woman, 34 years old).

Previous qualitative research has reported that patients felt coerced into taking medicines [5], implying a power imbalance [83]. Doctors thus may serve as gatekeepers to whom patients may feel forced to subordinate to this power to get help (in the form of diagnosing and approval of sick-listing). As previously argued by Freidson, patients realizing that they need something from their doctor (for instance, sick-listing) must give in and accept what the doctors ‘suggests’, at least temporarily [82]. We must not forget that the clinical consultation is a transaction between two parties separated by differences in power, both social and symbolic [3], [84]. Patients have typically been submissive towards medical authority – accepting medical advice on trust because they lack the expertise to question it – often accepting a culture in which drugs are viewed as the appropriate remedy for a variety of ills [85]. Proponents of the medicalization critique draw attention to the notion that patients in general (because of their lack of medical knowledge) are placed in the position of vulnerable supplicants when they seek the attention of doctors and they have little opportunity to challenge doctors’ decisions [13]. It is necessary to distinguish between medicalization and medical dominance, however, which can be a part of medicalization but is not identical with it [14]. When doctors do not listen to their patients in the medical consultation and do not recognize their story, this is medical dominance in action; medicalization becomes the solution to the patients’ problems in terms of diagnosis and treatment.

#### Feeling abandoned by the doctor

Some patients described in the KILEN reports feeling abandoned by their doctor, sometimes throughout the entire treatment period. This could include a lack of follow-ups of treatment. Prescriptions were sometimes renewed without personal contact, for instance by telephone.

*While I have been medicating, my doctor and I have not spoken.* (Man, 56 years old).

According to the Swedish National Board of Health and Welfare, an evaluation of the effect of the prescribed antidepressant is the most important measure to minimize risks. The treatment should be reviewed on a regular basis so that the patient does not continue to take a drug without clear indication [41]. According to a study of antidepressant medication in primary care, however, the agency found that only 40% of Swedish patients had a follow-up appointment and more than 60% of these had used antidepressant drugs for over a year [41]. Some patients reported that abandonment meant feeling that no one cared for them, for their health, for their future, and for their struggle to get back to a functioning life. A few patients even felt disrespected or ill-treated by their doctor not just during but also after antidepressant treatment.

*This one [the doctor] after I ended drug treatment has have been malicious and unpleasant and very unprofessional in his attitude towards me.* (Woman, 41 years old).

Swedish research has shown that patients with psychiatric disorders reported feeling wronged to a higher degree than patients with somatic disorders [86] and that feelings of doctors’ nonchalance and disrespect are powerful explanations as to why patients feel mistreated [87]. This may risk influencing the patient’s entire experience of the medical encounter in a negative way.

### Conclusions

As mentioned in the introduction, research suggests that nowadays a biomedical approach is downplayed in the medical encounter in favor of a patient-oriented perspective. The KILEN data suggests, however, that the dominance of the doctor, instead of a patient oriented perspective, strongly may affect the medical encounter. As indicated in the KILEN’s consumer reports (and other studies as well), doctors tend to individualize social problems in the medical encounter. The challenge for both doctors and patients is to mobilize medicalization when it is appropriate and to do so in a collaborative approach between doctor and patient rather than by medical dominance [14]. Middleton and Moncrieff, among others, argue that the patient, not the doctor, is the expert in the medical encounter, and the role of the doctor is to help and support patients in identifying the nature of their problems and the way to address them [88].

The issue is not that depression in itself is medicalized, however, because it has been so for quite some time. The main concern is rather that some doctors (1) quickly decide on a depression diagnosis without listening to what the patient has to say and (2) quickly decide on an antidepressant treatment strategy without considering alternatives. However, one must acknowledge also the increased pressure towards medicalization that may stem from the activities of certain social movements and interest groups [89]. Once regarded as passive victims of medicalization, patients now can hold vital positions as advocates, consumers, or even agents of change [90]. A diagnosis is becoming increasingly essential in order to get access to not only medical treatment but also to receive support within (for instance) the education system. Thus, aspects of consumerism, together with industry promotion, medicalization, and deregulatory state policies, are found to be drivers of pharmaceuticalization in ways that are largely outside (or suboptimal for) significant therapeutic advances in the interest of public health [58]. For the sake of public health, it is therefore crucial to patrol the boundaries of medicalization and especially the ones of pharmaceuticalization. Maybe we ought to ask ourselves if it is really the responsibility of the doctor and the health care system to handle everyday problems or whether people turn to these institutions because they have nowhere else to go. An emphasis on pharmaceutical products may divert attention from not only other approaches to health care such as psychotherapy, illness prevention, and not least general public health interventions but also wider structural and political factors. A biochemical understanding of mental ill health may be embraced because it relieves people of responsibility for their circumstances, but relieving people of responsibility can also result in a sense of powerlessness. The magic bullet approach may have its merits but can also jeopardize treatment by failing to see ‘the big picture’. This can contribute to questionable medicalization and/or pharmaceuticalization of depression.
